# Recommendations for a core assessment set for neurological physiotherapy entry-level education in Austria - a multistage process including a Delphi study

**DOI:** 10.1186/s12909-025-07704-8

**Published:** 2025-08-05

**Authors:** Andrea Greisberger, Meike Klinger, Anna Dopona, Manuela Riegler, Veronika Müller, Agnes Wilhelm, Theres Wess, Annette Nägele, Hannes Aftenberger, Katharina Kurz, Barbara Seebacher

**Affiliations:** 1 Department Health Sciences, University of Applied Sciences Hochschule Campus Wien, Favoritenstrasse 232, Wien, 1100 Austria; 2 Department Health and Social Sciences, Carinthia University of Applied Sciences, Klagenfurt am Wörthersee, Austria; 3 University of Applied Sciences for Health Professions Upper Austria, Steyr, Austria; 4 Department Health Sciences, IMC University of Applied Sciences Krems, Krems an der Donau, Austria; 5 Department Health Studies, FH Joanneum GmbH, Graz, Austria; 6 Clinic for Rehabilitation Muenster, Department of Rehabilitation Science, Muenster, Austria; 7 Clinical Department of Neurology, Medical University of Innsbruck, Innsbruck, Austria

**Keywords:** Core assessment set, Neurological physiotherapy, Bachelor’s degree, Delphi technique, Neurology, Physical therapy specialty, Standardised assessments

## Abstract

**Background:**

Standardised assessments are essential for diagnosing conditions, evaluating therapy, and formulating prognoses within physiotherapy. However, due to various barriers, including lack of knowledge, accessibility issues, and time constraints their routine use remains inconsistent. Defining a core set of standardised assessments for entry-level education is crucial in addressing these challenges. This study therefore aimed to establish such a core assessment set for Austria’s bachelor’s degree programmes for neurological physiotherapy and develop corresponding educational materials.

**Methods:**

A multistage process (2019–2024) was applied: (1) definition of the scope of the core assessment set; (2) preparation of a modified Delphi process, including initial screening of recommended assessments; (3) development of the core set through a modified Delphi process; (4) categorisation of assessments according to the intended level of student engagement; and (5) creation of the necessary content and structure for educational material to facilitate implementation. Representatives of all nine Austrian bachelor’s programmes participated in the whole process. Assessments were categorised according to their intended level of student engagement: RECOGNISING (students should become familiar with the assessment through exposure) and APPLYING (students should learn how to perform and interpret the assessment). Voting results were analysed using descriptive statistics.

**Results:**

Based on a number of 102 assessments as recommended by the Academy of Neurologic Physical Therapy with 17 supplementary assessments included to infuse Austrian context, assessment screening and three Delphi rounds were administered. Results classified 15 assessments as RECOGNISING and 22 assessments as APPLYING. Nineteen assessments were placed on a supplementary list for further learning. Educational materials were developed to support students and educators.

**Conclusions:**

The consensus-based core assessment set for neurological physiotherapy education in Austria ensures a necessary consistency across bachelor’s degree programmes. The developed educational materials support implementation and can serve as a resource for both students and practitioners. Future efforts should focus on updating and refining the core set based on stakeholder feedback while monitoring its impact on clinical education and practice.

**Trial registration:**

COMET (Core Outcome Measures in Effectiveness Trials) Database https://www.comet-initiative.org/Studies/Details/2046.

**Supplementary Information:**

The online version contains supplementary material available at 10.1186/s12909-025-07704-8.

## Background

Neurological physiotherapy seeks to enhance the quality of life of individuals with neurological conditions by employing movement-based approaches [1, 2]. With each patient’s presentation of a neurological condition varying, treatment must be specifically tailored to patient requirements. The individualisation of the physiotherapeutic process begins with the initial patient examination, whereby standardised and non-standardised assessments support diagnosis, prognosis and any subsequent management plan [3]. Tradition within physiotherapy has utilised non-standardised or poorly standardised assessments, however the increasing adoption of standardised assessments is associated with an upcoming demand for implementation of evidence-based practice (EBP). Using standardised assessments within the physiotherapeutic process enhances communication with patients and supports the development of a plan of care [4]. However, studies have shown that standardised assessments are not yet used regularly in physiotherapy practice [5–8]. Their inclusion in physiotherapy education therefore remains crucial to promote future uptake and inclusion from the onset in the professional development of evidence-based practice.

The development of competencies for applying EBP, including selection and use of standardised assessments, begins during professional training [9–11]. Therefore, educational programmes should be designed specifically to address such competencies [12–15]. Currently, there are nine three-year educational programmes for physiotherapy (bachelor’s degree) in Austria that transitioned from vocational to academic education between 2006 and 2014 [16]. All educational programmes incorporate theoretical and clinical training units on the basis of national law [17, 18]. Theoretical training has to be linked to the respective university, whereas clinical training is to be associated with a hospital, rehabilitation centre, or outpatient institute anywhere in Austria or, providing that certain requirements are met, abroad. During clinical training, students are supervised and assessed by their internship instructor. Although clinical training must cover at least 25% of the whole education programme, the positioning of the clinical training components within the three-year bachelor programme is individually regulated within each curriculum. Universities and institutions for clinical training liaise at an institutional level and there is no imbursement for the institutions or the clinical educators. Consequently, besides legally regulated requirements for clinical training, universities do not have a profound influence on clinical training. Nevertheless, most universities organise regular meetings to foster exchange between lecturers and instructors.

Despite the introduction of EBP and the demand for the use of standardised assessments, numerous barriers hinder their comprehensive adoption into daily practice for physiotherapists and physiotherapy students. These barriers include a lack of time, which causes difficulties in identifying appropriate assessments, inadequate knowledge and attitudes of physiotherapists regarding standardised assessments and psychometric properties, and the limited availability of manuals in the appropriate language [4, 7, 19–23]. To overcome some of these obstacles, it has been suggested that defining a toolkit and establishing a core set could be helpful [24–26]. Familiarity with assessments is one of the key facilitators for assessments to be used in daily practice [22, 23, 27].

The aim of this study was to establish a core set of standardised assessments for entry-level neurological physiotherapy education in Austria and to develop educational material to support their implementation and responsible use in both theoretical and clinical education.

## Methods

### Design

The multistage process (2019–2024) included (1) definition of the scope of the core assessment set; (2) preparation of a modified Delphi process, including an initial screening of recommended assessments from the Academy of Neurologic Physical Therapy (ANPT); (3) development of the core assessment set to reach expert consensus by applying a modified Delphi process [28, 29]; (4 ) categorisation of assessments according to the intended level of student engagement; and (5) creation of the necessary content and structure for educational material to support the implementation of the core assessment set across all bachelor’s degree programmes in Austria. All nine Austrian bachelor’s degree programmes remained involved throughout the process.

The project was registered in March 2022 with the repository of the Core Outcome Measures in Effectiveness Trials (COMET) initiative [30]. The methods and results of this manuscript are reported in accordance with the Core Outcome Set-STAndards for Reporting (COS-STAR) [31]. The study was conducted in accordance with the Declaration of Helsinki [32].

### Definition of the scope of application of the core assessment set

From 2019 to 2020, the Austrian network of neurological physiotherapy in higher education (Österreichisches Hochschulnetzwerk Physiotherapie in der Neurologie, ÖHPN) reached a consensus on core neurological diagnoses and symptoms to be included in entry-level physiotherapy education by an initial brainstorming process and applying a nominal group technique [33]. This consensus aimed to clearly define the scope of application for the core assessment set. Lecturers from all bachelor’s degree programmes prepared an initial list of symptoms and diagnoses taught in neurologic physiotherapy education. They focused on symptoms and diagnoses where the whole physiotherapeutic process from examination until evaluation is trained and taught theoretically and practically at the universities. On the basis of this list, the scope of the application for the core assessment set was discussed. After the discussion lecturers voted which of these symptoms and diagnoses should be the scope for the core set. A 70% agreement was considered to be a consensus for inclusion. A subsequent online discussion invited lecturers to reflect upon and discuss the decision.

Furthermore, a classification system for assessments was devised by drawing upon recommendations for standardised assessments in entry-level physiotherapy from the Academy of Neurologic Physical Therapy (ANPT) in the United States of America [34]. The ANPT categorised assessments as either ‘students should learn to use’ or ‘students should be exposed to’. This framework was subsequently tailored to suit the Austrian context.

### Preparation of the modified Delphi process

After the scope of application of the core set was defined, a small working group was built to plan and monitor the subsequent process. The working group consisted of five people: AG, AW, BS, MR and VM. The working group members ranged in age from 31 to 53 years (mean 40; standard deviation [SD] 8) and had between 10 and 32 years of experience as physiotherapists (mean 19; SD 8). Additionally, group members have been involved in teaching bachelor’s degree programmes for between 3 and 13 years (mean 8; SD 3).

#### Delphi panel

One designated representative was appointed from each bachelor’s degree programme to liaise with the working group, responsible for communicating the collective position of their respective programme. Age and years of experience as a physiotherapist were recorded for each representative (wording of the questions see additional file 1). The criteria for nomination as a representative included holding a faculty position within a degree programme and possessing at least one year of teaching experience in the field of neurological physiotherapy. Consequently, a maximum of nine votes per step was established, corresponding to the nine bachelor’s degree programmes in Austria. These programmes vary substantially across Austria in terms of the number of students and, subsequently, in the availability of full-time and part-time lecturers: in some locations only one person teaches neurological physiotherapy, other locations may have up to five lecturers in this area. The decision of who would be nominated as a representative was made at each location to meet the organisational needs of each study programme. To ensure active participation and inclusive discussions, all lecturers of the ÖHPN were invited to engage in online or face-to-face discussions and communicate with the representatives.

Each step was scored via an Excel-spreadsheet, distributed to the designated representatives, who subsequently returned it to AG to collate and summarise the results, ensuring that all involved parties remained blinded to individual scoring.

#### Compilation of the voting list for the Delphi process

The initial voting list included recommended assessments for entry-level physiotherapists from the ANPT [34], supplemented with assessments for diagnoses and symptoms from the scope of the application of the core assessment set not included in these recommendations. All assessments were categorised according to the International Classification of Functioning, Disability and Health (ICF) [35] by MR, VM, and BS. In instances of discrepancies, discussions within the working group were held, and consensus was sought. The categorisation mainly followed Schädler et al. [36], which is a comprehensive compendium of nearly 100 assessments in the German language that could be used in neurologic physiotherapy. Each assessment is described according to its background/development, validity, reliability, and responsiveness. Furthermore, recommendations regarding whether this assessment should be used for diagnosis, prognosis and/or evaluation are given. If not listed by Schädler et al. [36], the procedure suggested by Cieza et al. [37] was applied, linking the concepts within each item and response options of the assessment to the corresponding ICF chapter. Furthermore, each assessment was supplemented with details on the administration time, licencing requirements, costs, and links for further information. Both the ICF classification and information on practicability were documented in the Excel-spreadsheet used for scoring during subsequent screening and the Delphi process.

#### Screening

It was anticipated that not all assessments relevant for entry-level education in the United States would be applicable to the Austrian context due to differences in the healthcare system and in the responsibilities entry-level physiotherapists have within these systems. Therefore, the relevance of each assessment within the Austrian context underwent an initial individual and blinded screening by the working group members via a 4-point Likert scale (1 = very relevant to 4 = irrelevant) (December 2021). Assessments receiving ratings of 3 or 4 from all group members were excluded. The resulting list served as the foundation for further screening by all nine Austrian bachelor’s degree programmes via the same procedure (May 2022). In this step additional relevant standardised assessments deemed necessary for the Austrian context could be named (for the wording of the questions see additional file 1). Additionally, representatives were invited to express any preferences regarding future voting categories.

### Modified Delphi process

The subsequent Delphi rounds were structured into two phases: First, an anonymous survey using an Excel-spreadsheet was conducted, followed by an online discussion of the votes and communication of the next steps.

#### First Delphi round

In the first Delphi round, the following questions were asked: *Should assessment X be included in a core assessment set in entry-level neurological physiotherapy education?* Initially, only three response categories were proposed: (1) the assessment should be included in entry-level neurological physiotherapy education, (2) unclear and (3) the assessment should be excluded from entry-level physiotherapy education. However, on the basis of the feedback received after the screening process, a fourth category (assessment should be included on a supplementary list) was added. The responses were summarised and analysed according to a predefined scheme (see Table 1).

**Table 1:** Defining consensus in the Delphi process

Summarised voting results	Consensus
**FIRST DELPHI ROUND**	
≥ 70% - assessment should be included ≤ 15% - assessment should be excluded	Inclusion in core assessment set
≥ 70% - assessment should be excluded ≤ 15% - assessment should be included	Exclusion from core assessment set
≥ 70% - assessment should be included on a supplementary list	Supplementary list
All other results	Discussion
**SECOND DELPHI ROUND**	
≥ 70% - assessment should be included	Inclusion in core assessment set
≥ 70% - assessment should be excluded or added on a supplementary list	Supplementary list or exclusion from core assessment set
**THIRD DELPHI ROUND**	
≥ 50% - assessment should be included	Inclusion in core assessment set
≥ 50% - assessment should be excluded or added on a supplementary list	Supplementary list or exclusion from core assessment set

The second step of the first Delphi round involved an online discussion via ZOOM (June 2022), with a virtual wall (Padlet) [38] serving as an additional communication platform during and after the meeting. Prior to the discussion, the voting results were anonymously sent via email to the representatives of the bachelor’s degree programmes. All lecturers were invited to participate in the online meeting during which the voting results were summarised and discussed. To inform the subsequent Delphi round, lecturers were encouraged to share arguments for the inclusion or exclusion of specific assessments on the Padlet. Additionally, specific questions, e.g. on psychometric properties, could be posted on the Padlet, which were answered by BS and AG.

#### Second and third Delphi rounds

The second Delphi round commenced with another anonymous voting session, during which the remaining assessments were categorised into one of two groups: (1) the assessment should be included in entry-level neurological physiotherapy education, and (2) the assessment should be excluded or added to a supplementary list. Like the first Delphi round, the voting results were communicated anonymously to the representatives. An online discussion via ZOOM was conducted to delve into the results and outline the subsequent decision-making process (April 2023). Discussions during this meeting, as well as additional discussions in all bachelor’s degree programmes, were again structured and summarised on a Padlet. This included listing all remaining assessments alongside the results of the second Delphi round voting. Lecturers were encouraged to post statements if they disagreed with the simple majority (≥ 50%) of the voting of the second Delphi round. These statements formed the basis for the third Delphi voting (May 2023). In this round, as in the second Delphi round, assessments were classified into two groups.

### Categorisation of assessments

A prefinal categorisation into the following four categories was performed individually and in a blinded manner by the members of the working group in June 2023: (1) students should become familiar with the assessment through exposure (RECOGNISING), (2) students should learn how to perform and interpret the assessment (APPLYING), (3) supplementary list, or (4) exclusion from the core assessment set. Assessments that did not reach 100% agreement among members of the working group were rediscussed twice and assigned one of the four categories. The compilation resulting from this process (prefinal categorisation) was communicated to the lecturers and finalised during a face-to-face meeting in September 2023 (final categorisation).

### Development of educational material for implementation

To facilitate the implementation of the core assessment set, lecturers expressed their need for specific educational materials. These materials should focus on concise information relevant to the practical use of the assessments, such as standardisation, scoring, and selected indicators of psychometric properties. Furthermore, these materials should support critical reflection by lecturers and students on the interpretation and limitations of standardised assessments. The content of the educational material was developed through several iterative cycles and through communication between the working group and lecturers. The methods for the literature search, screening, and data extraction were collaboratively developed by the working group. This part of the process was not included in the Delphi process.

### Data analysis

Descriptive statistics were performed to characterise the demographic attributes of the representatives. Furthermore, voting results from the two screenings, the three Delphi rounds and the prefinal categorisation were reported. Consensus was determined via predefined percentage thresholds (see Table 1). All calculations and summary statistics were performed using Microsoft Excel.

## Results

### Definition of the scope of application for the core assessment set

The brainstorming session initially yielded 44 different diagnoses in the field of neurological physiotherapy, categorised into pathologies affecting the central nervous system, the peripheral nervous system, and neuromuscular diseases. After discussion among 15 lecturers from every Austrian bachelor’s degree programme, a consensus was reached to focus on diseases and symptoms of the central nervous system as the scope of application for the core assessment set. This decision was made owing to the challenges in developing a consensus for diseases and symptoms related to the peripheral nervous system and neuromuscular diseases, which are covered in various other courses within the bachelor’s degree programmes. After anonymous voting and discussion, where each bachelor’s degree programme had one vote, the following diseases and symptoms were defined as the scope for the core assessment set: stroke, Parkinson’s disease, multiple sclerosis, traumatic brain injury, spinal cord injury, facial nerve paralysis, and ataxia. In a subsequent online-discussion concerns about the absence of loss of consciousness from the scope for the core set arose. It was agreed that disorders of consciousness should be embedded into the teaching of traumatic brain injury.

Building upon previous work [34], lecturers decided to classify assessments within the core assessment set in the following categories: (1) RECOGNISING: students are familiar with the assessment through exposure and (2) APPLYING: additionally, students can appropriately select, perform, and interpret the assessment in specific situations, including administering it with the use of manuals if necessary. RECOGNISING can be categorised as a learning outcome at levels 1 and 2 according to Bloom’s taxonomy of cognitive learning outcomes [39]. APPLYING can be classified as levels 1–4 according to Bloom [39] and as levels 1 and 2 according to Dave’s taxonomy of psychomotor learning outcomes [40]. Following the implementation of the core assessment set within each university’s theoretical training, students will be evaluated on their familiarity with assessments categorised under RECOGNISING and with their ability to select, perform, and interpret the assessment categorised under APPLYING as part of the learning objectives assessment.

### Preparation of the modified Delphi process

**Fig. 1:**
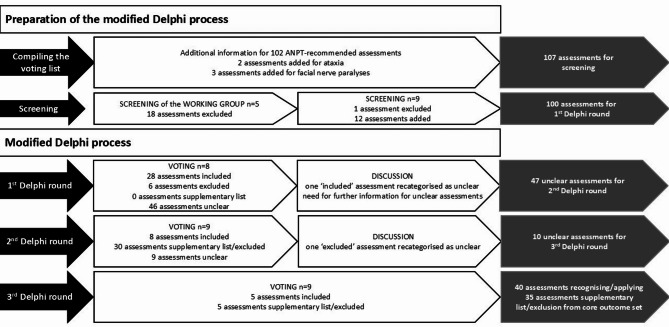
Summary of the results from the preparation of the Delphi process and the modified Delphi process itself. Legend: ANPT = Academy of Neurologic Physical Therapy in the United States of America, MS = multiple sclerosis, PD = Parkinson’s disease, SCI = spinal cord injury, TBI = traumatic brain injury

The preparation and realisation of the Delphi process, along with the results of each step, are summarised in Fig. 1.

#### Delphi panel

**Fig. 2:**
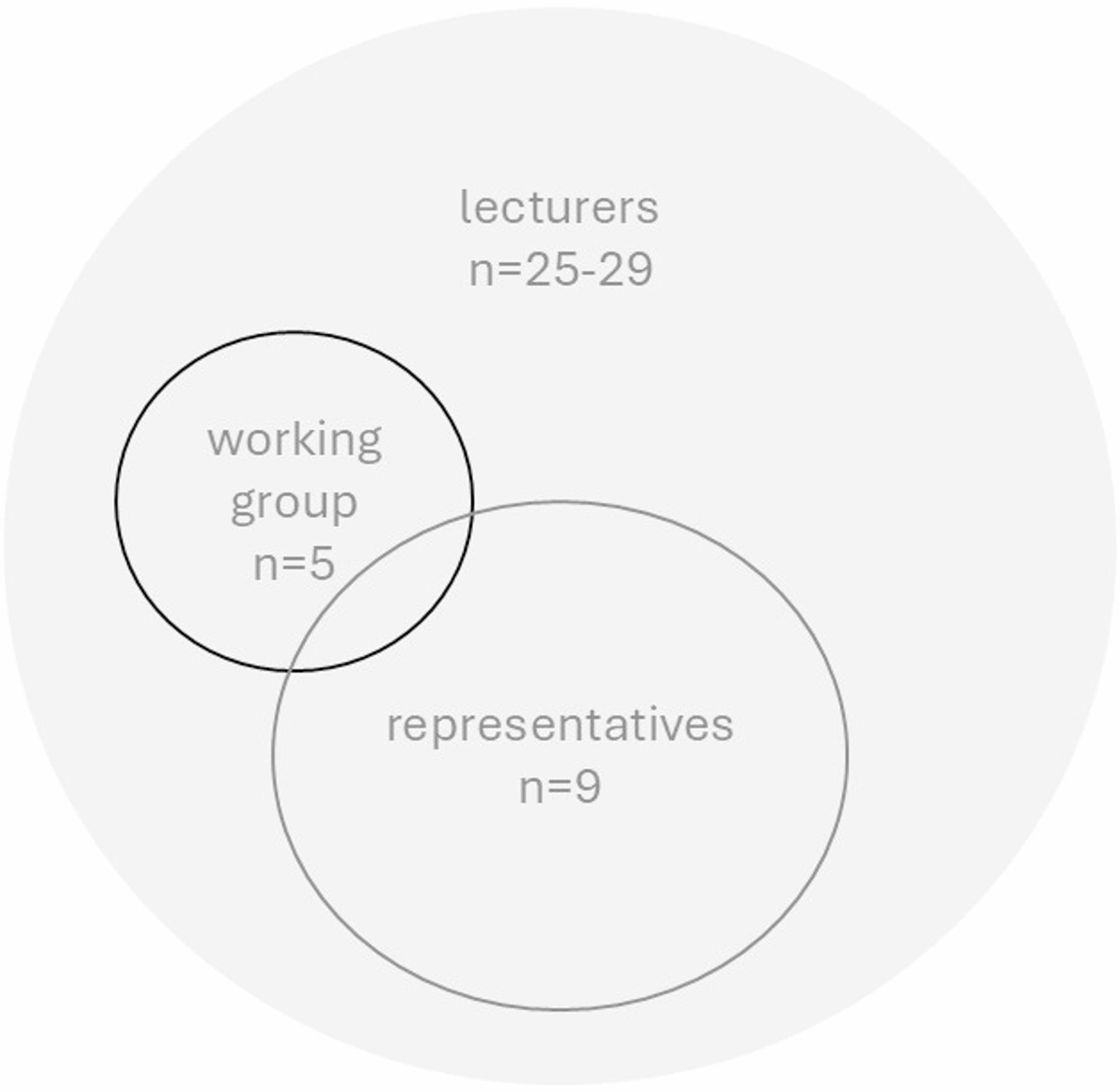
Roles within the project

The assigned representatives ranged in age from 30 to 51 years (mean 39; SD 7.5) with between 8 and 27 years of experience as physiotherapists working with neurological patients (mean 15.9; SD 6.8). All, aside one who practiced as a physiotherapist until 2018, were actively working as physiotherapists alongside their academic position within the university. Two members of the working group (MR and VM) were also representatives of a bachelor’s degree programme. Owing to staff changes between 2021 and 2023, there was a change in the representative for one bachelor’s degree programme during the process. One representative was unable to participate in the first Delphi voting due to sick leave. As she was the only lecturer in neurology within this bachelor’s degree programme no substitute could be sent. Nonetheless, she retrospectively consented to the summarised results of this round. Figure 2 graphically describes the different (overlapping) roles within the project.

#### Compilation of the voting list for the Delphi process

The development of the core assessment set was based on 102 recommended assessments for entry-level physiotherapists from the ANPT [34]. However, since the ANPT does not recommend standardised assessments for the symptoms of ataxia and facial nerve paralysis, additional assessments were identified through a comprehensive literature search conducted by AW. Specifically, the Scale for the Assessment and Rating of Ataxia (SARA) and the International Cooperative Ataxia Rating Scale (ICARS) were added to assess the symptom of ataxia. For facial nerve paralysis, the Sunnybrook Facial Grading Scale, Facial Clinimetric Evaluation Instrument, and the Facial Nerve Grading Scale 2.0 were included.

#### Screening

The initial screening process performed by the working group reduced the number of assessments to 89. In the subsequent screening involving all nine bachelor’s degree programmes, 12 assessments relevant to the Austrian context were added, while one assessment was deemed irrelevant, resulting in a total of 100 assessments for the first Delphi voting. Detailed results of the screening process can be found online in the supplementary material to this article (see additional file 2).

### Modified Delphi process - first, second, and third Delphi rounds

These 100 assessments formed the basis for the first Delphi round. After voting, 28 assessments were categorised as ‘should be included in entry-level physiotherapy education’, 26 assessments were excluded (see Table 2 for voting results for these assessments) and 46 remained unclear. One assessment (maximal inspiratory/expiratory pressure) was initially categorised as ‘assessment should be included in entry-level physiotherapy education’. However, the discussion after the voting revealed substantial costs related to measuring inspiratory/expiratory pressure. Furthermore, very few internships within neurological physiotherapy provide the opportunity to gain experience in measuring inspiratory/expiratory pressure. Therefore, this assessment was re-categorised as unclear, resulting in 47 assessments for the second Delphi round.

**Table 2:** Assessments with final decision in the 1st Delphi round

Assessment	1st Delphi round *n* = 8			
	# of votes Inclusion	# of votes Unclear	# of votes Exclusion	# of votes Supplementary list
**Included Assessments**				
6-Minute Walk Test	8	0	0	0
10-Metre Walk Test	8	0	0	0
ASIA Impairment Scale	8	0	0	0
Barthel Index	8	0	0	0
Berg Balance Scale	8	0	0	0
Borg Scale	8	0	0	0
Dynamic Gait Index	8	0	0	0
Five Times Sit to Stand Test	8	0	0	0
Functional Reach Test	8	0	0	0
Glasgow Coma Scale	8	0	0	0
Manual Muscle Test	8	0	0	0
Modified Ashworth Scale	8	0	0	0
Modified Tardieu Scale	8	0	0	0
Numeric Pain Rating Scale	8	0	0	0
Scale for the Assessment and Rating of Ataxia (SARA)	8	0	0	0
Timed Up and Go	8	0	0	0
9-Hole Peg Test	7	0	0	1
Dynamometry	7	0	1	0
Freezing of Gait Questionnaire	7	0	1	0
Functional Ambulation Category	7	0	1	0
Goal Attainment Scaling	7	0	0	1
MDS-UPDRS Part I-III	7	0	1	0
Mini Balance Evaluation Systems Test (Mini BESTest)	7	1	0	0
POMA	7	0	1	0
Trunk Control Test	7	1	0	0
Fugl-Meyer Assessment of Motor Performance (FMA)	6	1	1	0
Timed Up and Go motor	6	0	1	1
**Excluded Assessments**				
Balance Error Scoring System	0	0	8	0
Facial Clinimetric Evaluation Instrument (FaCE)	0	0	8	0
Neurological Outcome Scale for Traumatic Brain Injury (NOS-TBI)	0	0	8	0
Balance Evaluation Systems Test (CB&M)	0	1	7	0
Community Integration Questionnaire I (CIQ I)	0	1	7	0
Disorders of Consciousness Scale	0	1	7	0
Facial Nerve Grading Scale 2.0	0	0	7	1
Glasgow Outcome Scale-Extended (GOSE)	0	1	7	0
International Cooperative Ataxia Rating Scale (ICARS)	1	0	7	0
Multiple Sclerosis Functional Composite (MSFC)	1	0	7	0
Patient Health Questionnaire	0	1	7	0
Parkinson’s Disease Questionnaire-8 (PDQ-8)	1	0	7	0
Satisfaction with Life Scale	1	0	7	0
Trunk Control Measurement Scale	0	0	7	1
5-item EuroQoL (EQ-5D-5 L)	1	1	6	0
12-Item MS Walking Scale	0	0	6	2
Activities-Specific Balance Confidence Scale (ABC Scale)	1	1	6	0
Canadian Occupational Performance Measure (COPM)	1	1	6	0
Disabilities of the Arm, Hand & Shoulder Questionnaire (DASH)	0	1	6	1
Disability Rating Scale	0	2	6	0
Function in Sitting Test	1	1	6	0
Functional Assessment Measure	0	1	6	1
Functional Status Examination	1	1	6	0
Modified Fatigue Impact Scale	1	0	6	1
Penn Spasm Frequency Scale	0	1	6	1

Assessments with higher levels of agreement are listed higher in the list. 70% agreement equals 6 votes

ASIA = American Spinal Injury Association, MDS-UPDRS Part I to III = Movement Disorder Society-revision of the Unified Parkinson’s Disease Rating Scale-Part 1 to 3, POMA = Performance Oriented Mobility Assessment (Tinetti Test),

After the second Delphi voting, 9 assessments remained unclear. In the subsequent discussion, lecturers recognised that no assessment for facial nerve paralysis remained on the list. Therefore, the Sunnybrook Facial Grading Scale was recategorised as unclear and included among the assessments to be voted on in the third Delphi round. For the detailed voting results of the second Delphi round see Table 3 and Table  4.

**Table 3:** Assessments with final decision in the 2nd Delphi round

Assessment	1st Delphi round *n* = 8				2nd Delphi round *n* = 9	
	# of votes Inclusion	# of votes Unclear	# of votes Exclusion	# of votes Supplementary list	# of votes Inclusion	# of votes Exclusion / supplementary list
**Included Assessments**						
Scale for Contraversive Pushing	5	0	2	1	8	1
Fatigue Severity Scale	6	0	2	0	7	2
Functional Gait Assessment	5	0	3	0	7	2
2-Minute Walk Test	4	0	2	2	6	3
Freezing of Gait Score	6	0	2	0	6	3
SCIM	3	1	2	2	6	3
Timed Up and Go cognitive	5	0	1	2	6	3
Trunk Impairment Scale	3	2	1	2	6	3
**Excluded Assessments**						
12-Minute Walk/Run	2	1	5	0	0	9
CMSA	1	3	2	2	0	9
NeuroQoL	1	1	5	1	0	9
FSMC	2	0	5	1	1	8
MPI-SCI	0	2	5	1	1	8
PASS	1	3	3	1	1	8
Parkinson’s Fatigue Scale	2	0	5	1	1	8
RNLI	2	1	4	1	1	8
Static Standing Balance Test	2	0	6	0	1	8
STREAM	1	1	4	2	1	8
Walking While Talking	2	0	6	0	1	8
Bells and Star Cancellation Test	3	1	3	1	2	7
High-level Mobility Assessment	2	1	5	0	2	7
Modified Rankin Scale	1	0	5	2	2	7
Motor Activity Log	2	3	3	0	2	7
MSIS-29	1	0	5	2	2	7
PDQ-39	2	0	4	2	2	7
Rivermead Motor Assessment	2	1	4	1	2	7
Stroke Impact Scale 2.0	2	3	1	2	2	7
Visual Analog Scale Fatigue	5	0	2	1	2	7
BESTest	3	0	5	0	3	6
Coma Recovery Scale– Revised	3	1	4	0	3	6
Dizziness Handicap Inventory	1	1	4	2	3	6
Four Square Step Test	2	0	5	1	3	6
Maximum Oxygen Uptake	5	0	3	0	3	6
MIP / MEP	6	1	1	0	3	6
Motricity Index	5	0	2	1	3	6
NAS	2	0	4	2	3	6
SF-36	3	1	3	1	3	6
WISCI II	2	2	2	2	3	6

Assessments with higher levels of agreement are listed higher in the list. 70% agreement equals 6 votes

BESTest = Balance Evaluation Systems Test, CMSA = Chedoke-McMaster Stroke Assessment, FSMC = Fatigue Scale for Motor & Cognitive Functions, MIP/MEP = Maximal Inspiratory/Expiratory Pressure, MPI-SCI = Multidimensional Pain Inventory, SCI version, MSIS-29 = Multiple Sclerosis Impact Scale, NAS = Nottingham Assessment of Somato-sensation, NeuroQoL = Quality of Life in Neurological Disorders, PASS = Postural Assessment Scale for Stroke, PDQ-39 = Parkinson’s Disease Questionnaire-39, RNLI = Reintegration to Normal Living Index, SCI = Spinal cord injury, SCIM = Spinal Cord Injury Independence Measure, SF-36 = 36-Item Short Form Health Survey, STREAM = Stroke Rehabilitation Assessment of Movement, WISCI II = Walking Index for Spinal Cord Injury II

After the third Delphi voting (results see Table 4), 40 assessments were considered relevant for inclusion in the core assessment set, whereas 35 were classified as either irrelevant or added to a supplementary list.

**Table 4:** Assessments with final decision in the 3rd Delphi round

Assessment	1st Delphi round *n* = 8				2nd Delphi round *n* = 9		3rd Delphi round *n* = 9	
	# of votes Inclusion	# of votes Unclear	# of votes Exclusion	# of votes Supplementary list	# of votes Inclusion	# of votes Exclusion / supple-mentary list	# of votes Inclusion	# of votes Exclusion / supple-mentary list
**Included Assessments**								
De Morton Mobility Index	3	3	1	1	4	5	8	1
FIM	4	0	2	2	5	4	8	1
Sunnybrook Facial Grading Scale	1	0	7	0	-	-	8	1
Wolf Motor Function Test	4	1	3	0	5	4	7	2
**Excluded Assessments**								
Rivermead Mobility Index	5	1	2	0	5	4	0	9
NIHSS	4	0	3	1	4	5	1	8
CTSIB	4	1	2	1	4	5	2	7
SPPB	3	2	2	1	4	5	3	6
Box and Blocks Test	3	0	3	2	5	4	4	5

Assessments with higher levels of agreement are listed higher in the list.

CTSIB = Clinical Test of Sensory Interaction in Balance, FIM = Functional Independence Measure, NIHSS = National Institutes of Health Stroke Scale, SPPB = Short Physical Performance Battery

### Categorisation of assessments

The initial categorisation into RECOGNISING, APPLYING, supplementary list or exclusion performed by the working group resulted in 100% agreement among the group members for 12 assessments, leaving 63 assessments for discussion. Following two meetings (working group), a consensus was reached on 23 assessments for APPLYING, 12 for RECOGNISING, 21 for the supplementary list, and 19 assessments to be excluded from the core assessment set. In the final approval process with lecturers, some assessments were recategorised, resulting in 22 APPLYING assessments, 15 RECOGNISING assessments, and 19 assessments for the supplementary list (see Table 5). Final approval for this categorisation was granted by all nine representatives. Furthermore, it was decided that the core assessment set should be revised no later than 2034.

**Table 5:** Results of the final categorisation

	Voting of Working group *n* = 5 prefinal categorisation			
Assessment	# votes APPLYING	# votes RECOGNISING	#votes Supplementary list	#votes Exclusion
**Final Categorisation APPLYING**				
10-Metre Walk Test	***5***	0	0	0
6-Minute Walk Test	***5***	0	0	0
Berg Balance Scale	***5***	0	0	0
Borg Scale	***5***	0	0	0
De Morton Mobility Index	1	***4***	0	0
Dynamometry	***4***	1	0	0
Fatigue Severity Scale	2	***3***	0	0
Five Times Sit to Stand Test	***5***	0	0	0
Freezing of Gait Questionnaire	***1***	4	0	0
Functional Ambulation Category	***3***	1	1	0
Functional Gait Assessment	***4***	1	0	0
Goal Attainment Scaling	***4***	1	0	0
Manual Muscle Test	***5***	0	0	0
Mini Balance Evaluation Systems Test (Mini BESTest)	***5***	0	0	0
Modified Tardieu Scale	***2***	3	0	0
Numeric Pain Rating Scale	***5***	0	0	0
Scale for Contraversive Pushing	***4***	1	0	0
Scale for the Assessment and Rating of Ataxia	***5***	0	0	0
Sunnybrook Facial Grading Scale	***3***	1	1	0
Timed Up and Go	***5***	0	0	0
Timed Up and Go cognitive	***3***	1	1	0
Trunk Control Test	***3***	1	1	0
**Final Categorisation RECOGNISING**				
9-Hole Peg Test	3	***2***	0	0
2-Minute Walk Test	***4***	1	0	0
American Spinal Injury Association Impairment Scale	2	***3***	0	0
Barthel Index	0	***5***	0	0
Box and Blocks Test	0	1	***4***	0
Fugl-Meyer Assessment of Motor Performance	1	***3***	0	1
Functional Independence Measure	0	***5***	0	0
Functional Reach Test	4	***1***	0	0
Glasgow Coma Scale	1	***3***	1	0
MDS-UPDRS Part I-III	0	***4***	1	0
Modified Ashworth Scale	4	***1***	0	0
Performance Oriented Mobility Assessment	***2***	1	1	1
Timed Up and Go motor	***3***	1	1	0
Trunk Impairment Scale	3	***1***	0	1
Wolf Motor Function Test	0	3	***2***	0
**Final Categorisation SUPPLEMENTARY LIST**				
36-Item Short Form Health Survey (SF-36)	0	0	***3***	2
Action Research Arm Test	0	2	***3***	0
Balance Evaluation Systems Test (BESTest)	0	0	***1***	4
Clinical Test of Sensory Interaction in Balance	0	0	***4***	1
Coma Recovery Scale– Revised	0	1	***3***	1
Dynamic Gait Index	0	4	***1***	0
Freezing of Gait Score	1	2	***1***	1
High-level Mobility Assessment	0	0	***2***	3
Modified Rankin Scale	0	0	***3***	2
Motor Activity Log	0	0	***3***	2
Motricity Index	1	0	***1***	3
Multiple Sclerosis Impact Scale (MSIS-29)	0	0	***3***	2
National Institutes of Health Stroke Scale	0	0	***3***	2
Parkinson’s Disease Questionnaire-39	0	1	***3***	1
Parkinson’s Fatigue Scale	0	0	***1***	4
Rivermead Mobility Index	0	0	***3***	2
Short Physical Performance Battery	0	0	***4***	1
Spinal Cord Injury Independence Measure	0	3	***2***	0
Stroke Impact Scale 2.0	0	0	***3***	2
Walking Index for Spinal Cord Injury II	0	1	***3***	1
**Final Categorisation EXCLUSION**				
12-Minute Walk/Run	0	0	3	***2***
Bells and Star Cancellation Test	0	0	1	***4***
Chedoke-McMaster Stroke Assessment	0	0	3	***2***
Dizziness Handicap Inventory	0	0	2	***3***
Fatigue Scale for Motor & Cognitive Functions	0	0	1	***4***
Four Square Step Test	0	0	1	***4***
Maximal Inspiratory/Expiratory Pressure	0	1	0	***4***
Maximum Oxygen Uptake	0	1	1	***3***
Multidimensional Pain Inventory, SCI version	0	0	1	***4***
Nottingham Assessment of Somato-sensation	0	0	1	***4***
Postural Assessment Scale for Stroke	0	0	2	***3***
Quality of Life in Neurological Disorders	0	0	1	***4***
Reintegration to Normal Living Index	0	1	1	***3***
Rivermead Motor Assessment	0	0	1	***4***
Static Standing Balance Test	0	0	2	***3***
Stroke Rehabilitation Assessment of Movement	0	0	0	***5***
Visual Analog Scale Fatigue	0	0	0	***5***
Walking While Talking	0	0	1	***4***

***Bold/italics*** = prefinal categorisation through working group

### Development of educational material for implementation

During the development of the educational material, it became evident that striking the optimal balance between providing too much and too little information is challenging. Summarised information on standardised assessments in books, e.g. Schädler et al. [36] and homepages such as https://strokengine.ca/en/assessments/ or https://www.sralab.org/rehabilitation-measures were reviewed and discussed within the working group and with lecturers. They expressed concern that the given information from these sources cannot be efficiently used by students during their education. Furthermore, the desire for concise information in German arose. Finally, the following content for educational material (datasheets) was agreed upon. For all assessments classified as RECOGNISING or APPLYING, a general overview of the assessment should be given, including its classification according to the ICF. Furthermore, a link to a translated and validated version of the assessment in German should be provided, if available. Where applicable, references to population-specific recommendations, on the basis of the work of the ANPT [34], were included. These references aim to raise awareness of the importance of selecting assessments appropriate to specific patient populations. To provide guidance on the purpose of the assessment with respect to whether it should be used for evaluation, diagnosis and/or prognosis, recommendations from Schädler et al. [36] were cited. These references aim to raise awareness of the importance of selecting assessments appropriate to specific patient populations and clinical contexts. Finally, practical information, e.g. instructions or equipment required for the application of the assessment, was added.

For each assessment categorised as APPLYING, an additional systematic literature search was conducted in Medline via PubMed in order to give details on relevant clinical parameters. The search strategy comprised three blocks, connected with ‘AND’: (1) a search string for the scope of the core assessment set, (2) a search string for psychometric properties based on a suggested precise search string by Terwee et al. [41], and (3) a search string for the specific assessment for which the datasheet was to be completed (see additional file 3). While a detailed review, including a critical appraisal, of psychometric properties was beyond the scope of the project, this systematic literature search was the basis for extracting data on minimal detectable change, minimal clinically important difference, measurement error and cut-off values for known groups. The content of the educational material is listed in Table 6.

**Table 6:** Content of the educational material

**General assessment description**	Area of assessment	• Classified according to ICF, e.g. d4 mobility (d450-c469 walking and moving)
	Recommendations	• Schädler et al. [36] • ANPT [34]
	Assessment type	• Performance based measure • Patient-reported outcome measure
	Administration	• Instruction • Scoring • Equipment required • Administration time • Training required • Cost
**Relevant clinical parameter**		• Minimal detectable change • Minimum clinically important difference • Measurement error • Cut-off values for known groups

Relevant clinical parameters are only described for APPLYING assessments

ANPT– Academy of Neurologic Physical Therapy, ICF– International Classification of Functioning Disability and Health

## Discussion

With involvement of all Austrian bachelor’s degree programmes, a core assessment set for entry-level neurological physiotherapy was developed. Twenty-two assessments were identified which students should be able to select, perform and interpret in specific clinical situations. They should be familiarised with at least 15 additional assessments. Furthermore, 19 assessments potentially relevant to neurological physiotherapy were included in a supplementary list. To facilitate the selection, application, and interpretation of this core assessment set by students and physiotherapists in the field of neurology, educational material was developed following a transparent and systematic approach. The developed core assessment set can be used during clinical training in institutions outside universities as a benchmark for students’ competencies wherever they complete their theoretical training.

Enhancing response rates in Delphi studies is challenging as the process is time consuming for the involved experts and lasts an extended period of time [42, 43]. Providing participants with a predefined list of assessments may have helped maintain engagement by reducing the cognitive demands of each round. Although additional assessments could be proposed in the early stages, the structured format of discussions likely contributed to clarity and efficiency, which are recognised facilitators of the Delphi methodology [28, 44]. In the present study, the anonymous voting process and subsequent open, trust-driven discussions seemed to support loyalty among lecturers and might have contributed to maintaining a high response rate. The discussions in the ZOOM meetings and on the Padlet encouraged debates and information exchange, whereas the anonymous voting enabled reflection. Discussions were influenced by the challenge of balancing the recommendations from the literature, the experiences students gain during their clinical training, the expectations of clinical educators, and the limited time resources within the three-year bachelor’s degree programme. The inclusion of 19 assessments in a supplementary list reflects this tension, providing a resource for advanced learning without overburdening curricula.

Importantly, the recommended standardised assessments are intended to support, not replace, clinical reasoning. Their use should always be embedded within patient-centred decision-making. Overreliance on such tools– especially without understanding their limitations– can lead to misapplication, particularly among less experienced clinicians. To address this, the educational materials include brief information on selected psychometric properties. While not exhaustive, these summaries aim to raise awareness of key aspects and encourage the thoughtful use of assessments in practice.

The developed core assessment set and educational materials could also serve as instruments for professional development among practicing physiotherapists seeking to update their skills. For this purpose, the Austrian professional physiotherapy association was informed about the project from the outset and provided financial support without intending to influence the outcome [45]. Given that the needs of practitioners might differ from those of students, focused implementation strategies will be necessary. Successful implementation strategies, among other factors, need to emphasise practitioners’ perception that using standardised assessments is meaningful [46]. Physio Austria addressed this issue by launching a project to disseminate the core assessment set within the Austrian physiotherapy community [47].

Most of the assessments classified as APPLYING are recommended in systematic reviews or clinical practice guidelines for general neurology [48, 49], stroke [50–56], Parkinson’s disease [57, 58], multiple sclerosis [50, 59], spinal cord injury [60], ataxia [61], traumatic brain injury [62] and facial nerve paralysis [63]. There are seven assessments classified as APPLYING that were not identified in the above listed systematic reviews or clinical practice guidelines: the De Morton Mobility Index, Manual Muscle Test, Modified Tardieu Scale, Numeric Pain Rating Scale, Scale for Contraversive Pushing, Timed Up and Go cognitive, and Fatigue Severity Scale.

The De Morton Mobility Index focuses more on aging than on neurological conditions [64, 65], therefore this assessment was not deemed specifically relevant to neurological physiotherapy during discussions among lecturers. However, because the De Morton Mobility Index is also valid and reliable in patients with neurological conditions [66–68] and is widely used for geriatric conditions, it was agreed to add it to the core assessment set. The Manual Muscle Test is a basic physiotherapeutic assessment that focuses on body function and has its limitations. However, particularly in the context of spinal cord injury and interdisciplinary cooperation in spasticity management using Botulinum toxin, it is essential for physiotherapists to be able to apply a standardised test to assess muscle strength. Due to this, its inclusion was granted. The lecturers extensively discussed the inclusion of the Modified Tardieu Scale versus the Modified Ashworth Scale. The decision in favour of the Modified Tardieu Scale was based on educational reasons: applying the Modified Tardieu Scale clearly demonstrates that pathological muscle tone depends not only on movement velocity but also on pre-existing passive range of motion. Furthermore, it was hypothesised that if students could complete the Modified Tardieu Scale, they would also be able to use the Modified Ashworth Scale. The Modified Ashworth Scale was categorised as RECOGNISING. This decision is supported by a recent review that recommended the use of the Tardieu Scale in order to assess spasticity [69]. Educational reasons also contributed to the inclusion of the Scale for Contraversive Pushing and the Timed Up and Go cognitive. The Scale for Contraversive Pushing may be helpful in capturing the essence of pushing, and the Timed Up and Go cognitive is a brief assessment based on the widely recognised Timed Up and Go, which addresses the consequences of dual tasks on activities. The decision to include the Fatigue Severity Scale, rather than the Modified Fatigue Impact Scale or Fatigue Scale for Motor and Cognitive Function suggested by the ANPT was influenced by its broad use in Austria. The Numeric Pain Rating Scale was included because it was deemed necessary to have at least one simple method to measure pain, even though it was acknowledged that capturing pain can be challenging in patients with neurological conditions [70].

For most of the assessments classified as APPLYING, the educational material provides information on how to interpret results obtained from patients. However, for the Borg Scale (Rating of Perceived Exertion), Goal Attainment Scale, Manual Muscle Test, and Sunnybrook Facial Grading Scale, no studies have reported relevant clinical parameters in the context of neurological diseases and symptoms. This represents an area of future research to assist clinicians in interpreting standardised assessments.

However, this study is not without limitations. The number of participants in the Delphi rounds was limited to nine representatives– one from each of Austria’s bachelor’s degree programmes in physiotherapy. While this number may appear to be low, it was intentionally chosen to ensure equal and balanced representation across all institutions offering entry-level physiotherapy education. Each representative consulted with their respective neurological physiotherapy teaching team, enabling broader input beyond the individual level. Additionally, two representatives were also part of the working group, thereby holding dual roles within the project. This dual involvement may have influenced their perspectives through more in-depth engagement with the topic during working group discussions. However, both individuals were fully aware of their dual responsibilities and managed them appropriately through clear role-based communication.

The core assessment set is derived from a consensus among lecturers; with other stakeholders such as clinical educators, students, clinicians, and patients not included in the process, as the study focused on academic representatives with specific expertise in curriculum design and educational feasibility. In addition, limited resources restricted broader participation. This may have introduced a one-sided perspective on standardised assessments. Future studies should explore the inclusion of these additional stakeholders to broaden the relevance and acceptance of the core assessment set. Furthermore, basing the consensus primarily on expert opinion may have overlooked current evidence from systematic research. This could have introduced bias related to individual experiences and institutional priorities. However, since most of the assessments are recommended by clinical practice guidelines or have been found in systematic reviews, these individual influences may be acceptable.

Finally, the educational material was developed on the basis of informal feedback from lecturers, without a systematic assessment of students’ needs. Future iterations could benefit from integrating student perspectives to enhance the utility of these resources.

## Conclusions

In conclusion, this core assessment set represents a significant step toward harmonising neurological physiotherapy education in Austria and can now be gradually included in theoretical training. This implementation should be evaluated in terms of the development of students’ competences. On this basis the educational material could be further developed. Additionally, the relevance and usefulness of the core assessment set in daily physiotherapeutic practice should be researched and if necessary, the core assessment set should be revised. The establishment of a shared framework promotes consistency and quality across training programmes, ultimately enhancing patient care. Future efforts should focus on keeping up-to-date and refining the core assessment set based on feedback from multiple stakeholders.

## Electronic supplementary material

Below is the link to the electronic supplementary material.

Supplementary Material 1

Supplementary Material 2

Supplementary Material 3

## Data Availability

The Excel spreadsheet used for analysing the data used in the current study is available from the corresponding author upon reasonable request.

## Declarations

### Clinical trial number

The project was registered in March 2022 with the repository of the Core Outcome Measures in Effectiveness Trials (COMET) initiative (Registration 2046, https://www.comet-initiative.org/Studies/Details/2046).

## Abbreviations

ANPT: Academy of Neurologic Physical Therapy
EBP: Evidence-based practice
ICARS: International Cooperative Ataxia Rating Scale
ICF: International Classification of Functioning, Disability and Health
ÖHPN: Österreichisches Hochschulnetzwerk Physiotherapie in der Neurologie (Austrian Higher Education Network for Physiotherapy in Neurology)
SARA: Scale for the Assessment and Rating of Ataxia
SD: Standard deviation
