# Supplementary material for: Recommendations for a core assessment set for neurological physiotherapy entry-level education in Austria - a multistage process including a Delphi study
Source: BMC Med Educ. 2025 Aug 5;25:1145. doi: 10.1186/s12909-025-07704-8 (PMC12323081; doi:10.1186/s12909-025-07704-8)
Supplement: Supplementary file 1 — Supplementary Material 1 [file 12909_2025_7704_MOESM1_ESM.docx]

**Supplemental Material: Questions for demographic data and screening, survey prior to Delphi discussions**

**Questionnaire for demographic data**

- How old are you?
  [Wie alt bist du?]
- How many years of experience do you have as a physiotherapist working with neurologic patients?
  [Wieviel Jahre arbeitest du bereits als Physiotherapeut:in mit neurologischen Patient:innen?]
- Are you currently practicing as a physiotherapist? If not, how many years have you not worked with patients?
  [Arbeitest du derzeit als Physiotherapeut:in mit Patient:innen? Falls nein, seit wie vielen Jahren arbeitest du nicht mehr mit Patient:innen]

**Questionnaire for Screening**

- Which assessments are relevant for entry-level neurological physiotherapy education in Austria? Please rate each assessment with 1 (very relevant) to 4 (irrelevant).
  [Welche Assessments sind für die Bachelorausbildung im Fachbereich Neurologie / ZNS für die festgelegten Pflichtinhalte relevant? Bitte bewerte jedes Assessment mit 1 (sehr relevant) bis 4 (=irrelevant).]
- Are there any assessments that are relevant for the Austrian context and are not yet on the list? If so, please note them here.
  [Gibt es Assessments die für den österreichischen Kontext relevant sind und sich noch nicht in der Liste befinden? Falls ja, bitte hier notieren.]

**Survey prior to the first Delphi discussion**

- Should assessment X be included in a core outcome set in entry-level neurological physiotherapy education? Please use one of the following categories for your rating:(1) the assessment should be included in entry-level neurological physiotherapy education, (2) unclear, (3) the assessment should be excluded from entry-level physiotherapy education (4) the assessment should be added on a supplementary list.
  [Sollte das Assessment X in den Assessmentpool für den Fachbereich Neurologie /ZNS in die Bachelorausbildung aufgenommen werden? Antworte mit einer der folgenden Kategorien: (1) das Assessment sollte aufgenommen werden, (2) unklar, (3) das Assessment sollte nicht aufgenommen werden, (4) die Bewertung sollte in eine Liste mit ergänzenden Assessments aufgenommen werden.]

**Survey prior to the second and third Delphi discussion**

- Should assessment X be included in a core outcome set in entry-level neurological physiotherapy education? Please use one of the following categories for your rating:(1) assessment should be included, (2) assessment should be excluded or added on a supplementary list.
  [Sollte das Assessment X in den Assessmentpool für den Fachbereich Neurologie /ZNS in die Bachelorausbildung aufgenommen werden? Antworte mit einer der folgenden Kategorien: (1) das Assessment sollte aufgenommen werden, (2) das Assessment sollte nicht aufgenommen werden oder auf eine ergänzende Liste gesetzt werden.]
