# Supplementary material for: Recommendations for a core assessment set for neurological physiotherapy entry-level education in Austria - a multistage process including a Delphi study
Source: BMC Med Educ. 2025 Aug 5;25:1145. doi: 10.1186/s12909-025-07704-8 (PMC12323081; doi:10.1186/s12909-025-07704-8)
Supplement: Supplementary file 2 — Supplementary Material 2 [file 12909_2025_7704_MOESM2_ESM.docx]

**Supplemental Material: Results from the screening process**

| **Assessment** | **Working group**  **n=5** | | **Bachelor’s degree programmes**  **n=9** | |
| --- | --- | --- | --- | --- |
|  | **RELEVANT** | **IRRELEVANT** | **RELEVANT** | **IRRELEVANT** |
| **Excluded by screening of the working group** | | | | |
| Agitated Behaviour Scale | 0 | 5 | - | - |
| Arm Motor Ability Test | 0 | 5 | - | - |
| Assessment of Life Habits | 0 | 5 | - | - |
| Craig Handicap Reporting and Assessment Technique | 0 | 5 | - | - |
| Disease Steps | 0 | 5 | - | - |
| Functional Assessment of Multiple Sclerosis | 0 | 5 | - | - |
| Impact on Participation and Autonomy Questionnaire | 0 | 5 | - | - |
| Mayo Portland Adaptability Inventory–4 | 0 | 5 | - | - |
| Montreal Cognitive Assessment | 0 | 5 | - | - |
| Moss Attention Rating Scale | 0 | 5 | - | - |
| Multiple Sclerosis Quality of Life | 0 | 5 | - | - |
| Orientation Log | 0 | 5 | - | - |
| Quality of Life after Brain Injury | 0 | 5 | - | - |
| Rancho Levels of Cognitive Functioning- Revised | 0 | 5 | - | - |
| Sensory Organisation Test | 0 | 5 | - | - |
| Sickness Impact Profile | 0 | 5 | - | - |
| Sydney Psychosocial Reintegration Questionnaire | 0 | 5 | - | - |
| World Health Organization Quality of Life- BREF | 0 | 5 | - | - |
| **Excluded by screening of the bachelor’s degree programmes** | | | | |
| Capabilities of Upper Extremity Functioning Instrument | 1 | 4 | 0 | 9 |
| **Assessments for 1st Delphi round** | | | | |
| 10-Metre Walk Test | 5 | 0 | 9 | 0 |
| 12-Minute Walk/Run | 1 | 4 | 4 | 5 |
| 12-Item MS Walking Scale | 5 | 0 | 4 | 5 |
| 2-Minute Walk Test | 5 | 0 | 6 | 3 |
| 36-Item Short Form Health Survey (SF-36) | 2 | 3 | 5 | 5 |
| 5-item EuroQoL (EQ-5D-5L) | 2 | 3 | 4 | 5 |
| 6-Minute Walk Test | 5 | 0 | 9 | 0 |
| 9-Hole Peg Test | 4 | 1 | 8 | 1 |
| Action Research Arm Test | 3 | 2 | 6 | 2 |
| Activities-Specific Balance Confidence Scale | 4 | 1 | 4 | 5 |
| American Spinal Injury Association Impairment Scale | 5 | 0 | 8 | 1 |
| Balance Error Scoring System | 2 | 3 | 2 | 7 |
| Balance Evaluation Systems Test (BESTest) | 2 | 3 | 4 | 5 |
| Berg Balance Scale | 5 | 0 | 9 | 0 |
| Borg Scale - Rating of Perceived Exertion | 5 | 0 | 8 | 1 |
| Box and Blocks Test | 4 | 1 | 5 | 4 |
| Canadian Occupational Performance Measure | 2 | 3 | 2 | 7 |
| Chedoke-McMaster Stroke Assessment | 1 | 4 | 5 | 4 |
| Clinical Test of Sensory Interaction in Balance | 4 | 1 | 4 | 5 |
| Coma Recovery Scale – Revised | 3 | 2 | 5 | 4 |
| Community Balance and Mobility Scale | 1 | 4 | 2 | 7 |
| Community Integration Questionnaire I | 1 | 4 | 2 | 7 |
| Disabilities of the Arm, Hand & Shoulder Questionnaire | 3 | 2 | 4 | 5 |
| Disability Rating Scale | 1 | 4 | 2 | 7 |
| Disorders of Consciousness Scale | 1 | 4 | 1 | 8 |
| Dizziness Handicap Inventory | 4 | 1 | 3 | 6 |
| Dynamic Gait Index | 3 | 2 | 9 | 0 |
| Dynamometry | 5 | 0 | 7 | 2 |
| Facial Clinimetric Evaluation Instrument | 1 | 4 | 2 | 7 |
| Facial Nerve Grading Scale 2.0 | 3 | 2 | 2 | 7 |
| Fatigue Scale for Motor & Cognitive Functions | 2 | 3 | 4 | 5 |
| Five Times Sit to Stand Test | 5 | 0 | 9 | 0 |
| Four Square Step Test | 4 | 1 | 3 | 6 |
| Freezing of Gait Questionnaire | 5 | 0 | 7 | 2 |
| Fugl-Meyer Assessment of Motor Performance | 3 | 2 | 8 | 1 |
| Function in Sitting Test | 2 | 3 | 2 | 7 |
| Functional Ambulation Category | 5 | 0 | 7 | 2 |
| Functional Assessment Measure | 4 | 1 | 3 | 6 |
| Functional Gait Assessment | 5 | 0 | 5 | 4 |
| Functional Independence Measure | 4 | 1 | 5 | 4 |
| Functional Reach Test | 5 | 0 | 9 | 0 |
| Functional Status Examination | 1 | 4 | 2 | 7 |
| Glasgow Coma Scale | 5 | 0 | 8 | 1 |
| Glasgow Outcome Scale – Extended | 2 | 3 | 3 | 6 |
| High-level Mobility Assessment | 2 | 3 | 2 | 7 |
| International Cooperative Ataxia Rating Scale (ICARS) | 2 | 3 | 3 | 6 |
| Manual Muscle Test | 5 | 0 | 7 | 2 |
| Maximal Inspiratory/Expiratory Pressure | 2 | 3 | 7 | 2 |
| Maximum Oxygen Uptake | 1 | 4 | 6 | 3 |
| MDS-UPDRS Part I-III | 3 | 2 | 7 | 2 |
| Mini Balance Evaluation Systems Test (Mini BESTest) | 5 | 0 | 9 | 0 |
| Modified Ashworth Scale | 5 | 0 | 9 | 0 |
| Modified Fatigue Impact Scale | 5 | 0 | 4 | 5 |
| Modified Rankin Scale | 5 | 0 | 3 | 6 |
| Modified Tardieu Scale | 5 | 0 | 9 | 0 |
| Motricity Index | 5 | 0 | 8 | 1 |
| Multidimensional Pain Inventory, SCI version | 1 | 4 | 3 | 6 |
| Multiple Sclerosis Functional Composite | 1 | 4 | 4 | 5 |
| Multiple Sclerosis Impact Scale (MSIS-29) | 2 | 3 | 5 | 4 |
| National Institutes of Health Stroke Scale | 2 | 3 | 6 | 3 |
| Neurological Outcome Scale for Traumatic Brain Injury | 1 | 4 | 2 | 7 |
| Nottingham Assessment of Somato-sensation | 3 | 2 | 5 | 4 |
| Numeric Pain Rating Scale | 5 | 0 | 9 | 0 |
| Parkinson’s Disease Questionnaire-39 | 3 | 2 | 5 | 4 |
| Parkinson’s Disease Questionnaire-8 | 2 | 3 | 3 | 6 |
| Parkinson’s Fatigue Scale | 3 | 2 | 4 | 5 |
| Patient Health Questionnaire-9 | 1 | 4 | 2 | 7 |
| Penn Spasm Frequency Scale | 3 | 2 | 2 | 7 |
| Postural Assessment Scale for Stroke (PASS) | 3 | 2 | 3 | 6 |
| Quality of Life in Neurological Disorders (NeuroQoL) | 1 | 4 | 4 | 5 |
| Reintegration to Normal Living Index | 2 | 3 | 4 | 5 |
| Rivermead Mobility Index | 5 | 0 | 7 | 2 |
| Rivermead Motor Assessment | 3 | 2 | 4 | 5 |
| Satisfaction with Life Scale | 1 | 4 | 3 | 6 |
| Scale for the Assessment and Rating of Ataxia (SARA) | 5 | 0 | 9 | 0 |
| Spinal Cord Injury Independence Measure | 2 | 3 | 5 | 4 |
| Static Standing Balance Test | 2 | 3 | 4 | 5 |
| Stroke Impact Scale 2.0 | 2 | 3 | 6 | 3 |
| Stroke Rehabilitation Assessment of Movement | 2 | 3 | 5 | 4 |
| Sunnybrook Facial Grading Scale | 3 | 2 | 4 | 5 |
| Timed Up and Go | 5 | 0 | 9 | 0 |
| Timed Up and Go cognitive | 5 | 0 | 7 | 2 |
| Timed Up and Go motor | 5 | 0 | 7 | 2 |
| Trunk Control Test | 4 | 1 | 7 | 2 |
| Trunk Impairment Scale | 3 | 2 | 6 | 3 |
| Visual Analogue Scale Fatigue | 3 | 2 | 6 | 3 |
| Walking Index for Spinal Cord Injury II | 3 | 2 | 6 | 3 |
| Walking While Talking | 1 | 4 | 3 | 6 |
| **Assessments added by bachelor’s degree programmes** | | | | |
| Barthel Index | - | - | - | - |
| Bells Test and Star Cancellation Test | - | - | - | - |
| De Morton Mobility Index (DEMMI) | - | - | - | - |
| Fatigue Severity Scale | - | - | - | - |
| Freezing of Gait Score | - | - | - | - |
| Goal Attainment Scaling | - | - | - | - |
| Motor Activity Log | - | - | - | - |
| Performance Oriented Mobility Assessment (POMA) | - | - | - | - |
| Scale for Contraversive Pushing | - | - | - | - |
| Short Physical Performance Battery | - | - | - | - |
| Trunk Control Measurement Scale | - | - | - | - |
| Wolf Motor Function Test | - | - | - | - |

MDS – UPDRS Part I to III = Movement Disorder Society- revision of the Unified Parkinson's Disease Rating Scale – Part 1 to 3, SCI = Spinal cord injury

RELEVANT = score 1 & 2, IRRELEVANT score 3 & 4
