# Supplementary material for: Recommendations for a core assessment set for neurological physiotherapy entry-level education in Austria - a multistage process including a Delphi study
Source: BMC Med Educ. 2025 Aug 5;25:1145. doi: 10.1186/s12909-025-07704-8 (PMC12323081; doi:10.1186/s12909-025-07704-8)
Supplement: Supplementary file 3 — Supplementary Material 3 [file 12909_2025_7704_MOESM3_ESM.docx]

**Supplemental Material: Development educational material**

**Search strategy for the APPLYING assessments**

The following three search strings were combined with AND:

Search string related to assessments’ psychometric properties

((instrumentation[sh] OR Validation Study[pt] OR ‘‘reproducibility of results’’[MeSH Terms] OR reproducib*[ tiab] OR ‘‘psychometrics’’[MeSH] OR psychometr*[ tiab] OR clinimetr*[tiab] OR clinometr*[tiab] OR ‘‘observer variation’’[MeSH] OR observer variation[tiab] OR ‘‘discriminant analysis’’[MeSH] OR reliab*[tiab] OR valid*[tiab] OR coefficient[tiab] OR ‘‘internal consistency’’[ tiab] OR (cronbach*[tiab] AND (alpha[tiab] OR alphas[tiab])) OR ‘‘item correlation’’[tiab] OR ‘‘item correlations’’[ tiab] OR ‘‘item selection’’[tiab] OR ‘‘item selections’’[ tiab] OR ‘‘item reduction’’[tiab] OR ‘‘item reductions’’[tiab] OR agreement[tw] OR precision[tw] OR imprecision[tw] OR ‘‘precise values’’[tw] OR test–retest [tiab] OR (test[tiab] AND retest[tiab]) OR (reliab*[tiab] AND (test[tiab] OR retest[tiab])) OR stability[tiab] OR interrater[tiab] OR inter-rater[tiab] OR intrarater[tiab] OR intra-rater[tiab] OR intertester[tiab] OR inter-tester[tiab] OR intratester[tiab] OR intra-tester[tiab] OR interobserver[ tiab] OR inter-observer[tiab] OR intraobserver[tiab] OR intra-observer[tiab] OR intertechnician[tiab] OR intertechnician[ tiab] OR intratechnician[tiab] OR intra-technician[ tiab] OR interexaminer[tiab] OR inter-examiner[tiab] OR intraexaminer[tiab] OR intra-examiner[tiab] OR interassay[ tiab] OR inter-assay[tiab] OR intraassay[tiab] OR intra-assay[tiab] OR interindividual[tiab] OR inter-individual[ tiab] OR intraindividual[tiab] OR intra-individual[tiab] OR interparticipant[tiab] OR inter-participant[tiab] OR intraparticipant[tiab] OR intra-participant[tiab] OR kappa[tiab] OR kappa’s[tiab] OR kappas[tiab] OR ‘‘coefficient of variation’’[tiab] OR repeatab*[tw] OR ((replicab*[tw] OR repeated[tw]) AND (measure[tw] OR measures[tw] OR findings[tw] OR result[tw] OR results[tw] OR test[tw] OR tests[tw])) OR generaliza*[tiab] OR generalisa*[tiab] OR concordance[tiab] OR (intraclass[tiab] AND correlation*[ tiab]) OR discriminative[tiab] OR ‘‘known group’’ [tiab] OR ‘‘factor analysis’’[tiab] OR ‘‘factor analyses’’[tiab] OR ‘‘factor structure’’[tiab] OR ‘‘factor structures’’[tiab] OR dimensionality[tiab] OR subscale*[tiab] OR ‘‘multitrait scaling analysis’’[tiab] OR ‘‘multitrait scaling analyses’’[ tiab] OR ‘‘item discriminant’’[tiab]OR ‘‘interscale correlation’’[tiab] OR ‘‘interscale correlations’’[tiab] OR ((error[tiab] OR errors[tiab]) AND (measure*[tiab] OR correlat*[tiab] OR evaluat*[tiab] OR accuracy[tiab] OR accurate[tiab] OR precision[tiab] OR mean[tiab])) OR ‘‘individual variability’’[tiab] OR ‘‘interval variability’’[ tiab] OR ‘‘rate variability’’[tiab] OR ‘‘variability analysis’’[ tiab] OR (uncertainty[tiab] AND (measurement[tiab] 1122 Qual Life Res (2009) 18:1115–1123 123 OR measuring[tiab])) OR ‘‘standard error of measurement’’[ tiab] OR sensitiv*[tiab] OR responsive*[tiab] OR (limit[tiab] AND detection[tiab]) OR ‘‘minimal detectable concentration’’[tiab] OR interpretab*[tiab] OR (small*[tiab] AND (real[tiab] OR detectable[tiab]) AND (change[tiab] OR difference[tiab])) OR ‘‘meaningful change’’[tiab] OR ‘‘minimal important change’’[tiab] OR ‘‘minimal important difference’’[tiab] OR ‘‘minimally important change’’[tiab] OR ‘‘minimally important difference’’[tiab] OR ‘‘minimal detectable change’’[tiab] OR ‘‘minimal detectable difference’’[ tiab] OR ‘‘minimally detectable change’’[tiab] OR ‘‘minimally detectable difference’’[tiab] OR ‘‘minimal real change’’[tiab] OR ‘‘minimal real difference’’[tiab] OR ‘‘minimally real change’’[tiab] OR ‘‘minimally real difference’’[ tiab] OR ‘‘ceiling effect’’[tiab] OR ‘‘floor effect’’ [tiab] OR ‘‘Item response model’’[tiab] OR IRT[tiab] OR Rasch[tiab] OR ‘‘Differential item functioning’’[tiab] OR DIF[tiab] OR ‘‘computer adaptive testing’’[tiab] OR ‘‘item bank’’[tiab] OR ‘‘cross-cultural equivalence’’[tiab])) NOT ((‘‘address’’[Publication Type] OR ‘‘biography’’[Publication Type] OR ‘‘case reports’’[Publication Type] OR ‘‘comment’’[Publication Type] OR ‘‘directory’’[Publication Type] OR ‘‘editorial’’[Publication Type] OR ‘‘festschrift’’[ Publication Type] OR ‘‘interview’’[Publication Type] OR ‘‘lecture’’[Publication Type] OR ‘‘legal case’’[Publication Type] OR ‘‘legislation’’[Publication Type] OR ‘‘letter’’[Publication Type] OR ‘‘news’’[Publication Type] OR ‘‘newspaper article’’[Publication Type] OR ‘‘patient education handout’’[Publication Type] OR ‘‘popular work’’[Publication Type] OR ‘‘congress’’ [Publication Type] OR ‘‘consensus development conference’’[ Publication Type] OR ‘‘consensus development conference, nih’’[Publication Type] OR ‘‘practice guideline’’[ Publication Type]) NOT (‘‘animals’’[MeSH Terms] NOT ‘‘humans’’[MeSH Terms]) AND (address[Filter]) AND (address[Filter]))

Search string related to the scope of core outcome set

((((((((stroke) OR (parkinson)) OR (traumatic brain injury)) OR (spinal cord injury)) OR (multiple sclerosis)) OR (bell palsy)) OR (facial nerve paralysis)) OR ((stroke) AND (ataxia))) OR ((multiple sclerosis) AND (ataxia))

Search string related to the assessments

Common terms and abbreviations were used and connected with OR. For further information, please see the following table.

**Documentation of systematic searches for APPLYING assessments**

| **Assessment** | **Responsible person** | **Date** | **Search string for assessment** | **# records** | **# records for relevant clinical parameter** |
| --- | --- | --- | --- | --- | --- |
| 10-Meter Walk Test | Andrea Greisberger | 02/09/  2023 | (Ten-meter walking test[Title/Abstract]) OR (ten-meter walking[Title/Abstract]) OR (Ten-metre walking[Title/Abstract]) OR (Ten-metre walking test[Title/Abstract]) OR (10 m walk test[Title/Abstract]) OR (10 m walking test[Title/Abstract]) OR (10-m walking test[Title/Abstract]) OR (10-m walking[Title/Abstract]) OR (10MWT[Title/Abstract]) OR (timed 25-foot walk[Title/Abstract]) OR (timed 25 foot walk[Title/Abstract]) OR (T25FW[Title/Abstract]) OR (25FW[Title/Abstract]) | 606 | 29 |
| Five Times Sit to Stand Test | Anna Dopona, Andrea Greisberger | 13/12/2023 | (5 repetition sit to stand) OR (five repetition sit to stand) OR (five time sit to stand) OR (5 time sit to stand) OR (FTSTS) OR (5STS) OR (5xSTS) OR (5xSST) OR (5 repetition sit to stand test) OR (five repetition sit to stand test) OR (five time sit to stand test) OR (5 time sit to stand test) | 106 | 17 |
| 6-Minute Walk Test | Anna Dopona, Andrea Greisberger | 13/12/2023 | (6 minute walk test[Title/Abstract]) OR (6 minutes walk test[Title/Abstract]) OR (6 minute walking test[Title/Abstract]) OR (6 minutes walking test[Title/Abstract]) OR (6MWT[Title/Abstract]) OR (6-MWT[Title/Abstract]) OR (six minute walk test[Title/Abstract]) OR (six minutes walk test[Title/Abstract]) OR (six minute walking test[Title/Abstract]) OR (six minutes walking test[Title/Abstract]) OR (6 minute walk[Title/Abstract]) OR (6 minutes walk[Title/Abstract]) OR (6 minute walking[Title/Abstract]) OR (6 minutes walking[Title/Abstract]) OR (six minute walk[Title/Abstract]) OR (six minutes walk[Title/Abstract]) OR (six minute walking[Title/Abstract]) OR (six minutes walking[Title/Abstract]) | 389 | 29 |
| Berg Balance Scale | Andrea Greisberger | 28/04/2024 | berg balance scale[Title/Abstract] | 751 | 51 |
| De Morton Mobility Index | Anna Dopona | 21/03/2024 | (de Morton) OR (DEMMI) OR (de Morton Mobility Index)^[[1]](#footnote-1)^ | 61 | 4 |
| Dynamometry | Theres Wess | 22/08/2024 | (handheld myometer) OR (hand-held myometer) OR (hand-held myometry) OR (handheld myometer) OR (handheld dynamometer) OR (hand-held dynamometer) OR (hand-held dynamometry) OR (handhald dynamometer) | 66 | 4 |
| Fatigue Severity Scale | Theres Wess | 07/08/2024 | fatigue severity scale | 682 | 16 |
| Freezing of Gait Questionnaire | Agnes Wilhelm | 21/12/2024 | (FOGQ) OR (FOG Questionnaire) OR ("Freezing of Gait Questionnaire") OR (Freezing of Gait Questionnaire) | 208 | 2 |
| Functional Ambulation Category | Anna Dopona | 28/02/2024 | Functional Ambulation Category | 263 | 2 |
| Functional Gait Assessment | Anna Dopona | 20/03/2024 | (FGA[Title/Abstract]) OR (functional gait assessment[Title/Abstract]) | 72 | 10 |
| Goal Attainment Scaling | Katharina Kurz | 25/07/2024 | goal attainment scale | 126 | 0 |
| Manual Muscle Test | Hannes Aftenberger | 02/07/2024 | manual muscle test | 165 | 0 |
| Mini BESTest | Anna Dopona | 21/03/2024 | (miniBEST) OR (miniBESTest) OR (Mini-BESTest) OR (Mini-BEST) OR (mini BEST) OR (mini Balance Evaluation System Test) | 233 | 27 |
| Numeric Pain Rating Scale | Hannes Aftenberger | 02/07/2024 | "numeric rating scale" | 52 | 1 |
| Rate of Perceived Exertion (BORG Scale) | Annette Nägele, Andrea Greisberger | 01/11/2024 | (Borg) OR (borg scale) OR (rating of perceived exertion) OR ("rating of perceived exertion") OR (RPE) OR ("RPE") | 295 | 0 |
| Scale for Assessment and Rating of Ataxia | Agnes Wilhelm | 21/12/2024 | ("SARA scale") OR ("Scale for Assessment and Rating of Ataxia") OR (Scale for Assessment and Rating of Ataxia) OR (SARA) | 228 | 2 |
| Scale for contraversive Pushing | Manuela Riegler | 30/07/2024 | (Scale for Contraversive Pushing) OR (Clinical Scale for Contraversive Pushing) OR (Pusher Syndrome) OR (lateral pulsion) | 20 | 1 |
| Sunnybrook Facial Grading Scale | Katharina Kurz | 25/07/2024 | (Sunnybrook facial grading scale) OR (Sunnybrook facial grading system) | 91 | 0 |
| Tardieu Spasticity Scale (Modified Tardieu) | Anna Dopona | 10/04/2024 | (tardieu scale) OR (tardieu-scale) OR (modified tardieu scale) | 106 | 2 |
| Timed Up and Go | Anna Dopona | 10/04/2024 | "timed up and go"[Title/Abstract] | 704 | 38 |
| Timed Up and Go cognitive | Annette Nägele, Andrea Greisberger | 10/11/2024 | ((timed up and go) OR ("timed up and go") OR (TUG)) OR ("TUG")) AND ((dual task) OR (dual-task) OR (cognitive)) | 167 | 4 |
| Trunk Control Test | Manuela Riegler | 09/07/2024 | (trunk control test) OR (motricity index) OR (TCT) | 504 | 5 |

1. DEMMI – the literature search was conducted without search string for psychometric properties and for scope of core outcome set [↑](#footnote-ref-1)
